# Clonal Tracing of Heart Regeneration

**DOI:** 10.3390/jcdd9050141

**Published:** 2022-05-01

**Authors:** Kamal Kolluri, Taline Nazarian, Reza Ardehali

**Affiliations:** 1 Division of Cardiology, Department of Internal Medicine, David Geffen School of Medicine, University of California, Los Angeles, CA 90095, USA; kamal1470@g.ucla.edu (K.K.); talinenaz@g.ucla.edu (T.N.); 2 Eli and Edythe Broad Stem Cell Research Center, University of California, Los Angeles, CA 90095, USA; 3 Molecular, Cellular and Integrative Physiology Graduate Program, University of California, Los Angeles, CA 90095, USA; 4 Molecular Biology Institute, University of California, Los Angeles, CA 90095, USA

**Keywords:** lineage tracing, clonal expansion, proliferation, cardiac regeneration, cardiomyocyte proliferation, mosaic analysis with double markers, rainbow reporter

## Abstract

Cardiomyocytes in the adult mammalian heart have a low turnover during homeostasis. After myocardial injury, there is irreversible loss of cardiomyocytes, which results in subsequent scar formation and cardiac remodeling. In order to better understand and characterize the proliferative capacity of cardiomyocytes, in vivo methods have been developed to track their fate during normal development and after injury. Lineage tracing models are of particular interest due to their ability to record cell proliferation events over a long period of time, either during development or in response to a pathological event. This paper reviews two well-studied lineage-tracing, transgenic mouse models—mosaic analysis with double markers and rainbow reporter system.

## 1. Introduction

Heart disease is the leading cause of death worldwide and represents a major burden on healthcare costs [1,2,3,4]. As a terminally differentiated organ, the adult heart has limited regenerative capacity, and acute ischemic injury such as myocardial infarction (MI) often leads to replacement fibrosis [5,6,7]. Several studies have reported a limited proliferative capacity of cardiomyocytes (CM) in response to MI [8,9,10]. However, the origin of the newly generated cardiomyocytes, as well as the extent of regenerated myocardium, remains an area of controversy. As such, it is important to study the regenerative and proliferative capacity of cardiomyocytes in order to develop innovative therapies for patients suffering from ischemic heart disease.

The adult mammalian heart is considered to be a postmitotic organ, and various studies have estimated the annual turnover of cardiomyocytes to be around 1–1.5%, which declines with age [11,12]. Earlier methods utilized to study cardiomyocyte proliferation included tracking the expression of proliferation markers, or nucleotide incorporation [13,14,15,16,17]. Immunostaining for markers such as Ki67, phosphorylated histone H3 (PH3), and Aurora B kinase have been used in various studies, but staining for these markers can only reveal cardiomyocytes that are proliferating at a given point in time. This makes it difficult to detect the proliferative capacity of cardiomyocytes over time. In addition, these assays provide indirect surrogates for proliferation, especially in the context of cardiomyocyte polyploidy and potential DNA repair in response to injury. Unlike immunostaining, nucleotide incorporation studies such as Bromodeoxyuridine (BrdU) and 5-Ethynyl-2′-deoxyuridine (EdU) mark proliferating cells over a longer period of time, rather than providing a snapshot, which may offer a more accurate picture of cardiomyocyte proliferation. However, similar to immunostaining, these methods largely fail to distinguish karyokinesis from cytokinesis.

Lineage tracing is a method to track proliferating cells that circumvents some of the above concerns. Lineage tracing can be used to mark a finite number of cells at a specific time point and interrogate their role in normal development or their response to a certain pathological event [18]. Typically, a Cre recombinase gene is coupled with a tissue or cell-specific promoter, and upon Cre expression, loxP sites on a fluorescent reporter gene are excised, allowing expression of the fluorescent marker in all the target cells [19,20,21]. An equivalent Dre system relies on a Dre recombinase excising a pair of roxP sites and has been used both individually and in conjunction with the Cre system to produce dual recombinase systems [22,23]. Inducible lineage-tracing models using cell-specific markers can be designed for fate-mapping of target cells by indelibly labeling them with a fluorescent reporter. The reporter fluorescent label persists as a permanent genetic mutation, hence marking all the daughter cells. In the context of cardiac regeneration, lineage-tracing models can allow researchers to specifically label progenitors or cardiomyocytes and track their fate to examine their proliferation and their contribution to specific myocardial lineages. Due to permanent labeling, clonal analysis can allow researchers to track the localization of proliferating cells and determine whether the division is symmetric or asymmetric. Here, we review two lineage-tracing mouse models—mosaic analysis with double markers and rainbow—and their utility in studying cardiac regeneration.

## 2. Mosaic Analysis with Double Markers (MADMs)

### 2.1. Background and Methodology

**Figure 1:**
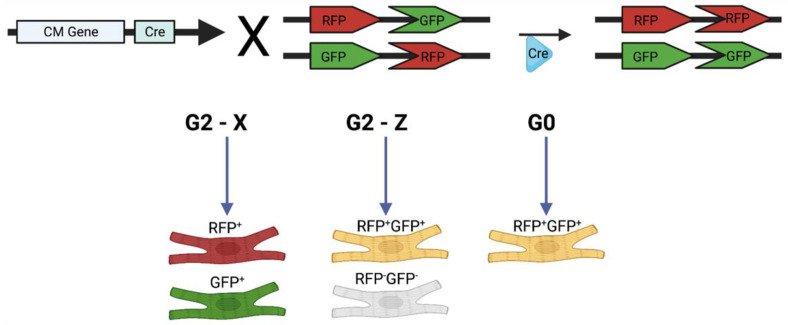
Schematic detailing how mosaic analysis with double markers (MADMs) works. In MADMs, the N-terminus of either the GFP or RFP allele, and the C-terminus of the other reporter, are knocked in on the same chromosome. After DNA replication, Cre/LoxP technology induces intrachromosomal recombination, and G2-X segregation generates two cells, one of which expresses RFP, and the other expresses GFP. G2-Z segregation results in 1 double-positive cell (yellow color) and 1 negative (no color) cell. Recombination in the G0 phase results in 1 double-labeled yellow cell. Created using BioRender.com, accessed on 29 March 2022.

Mosaic analysis with double markers (MADMs) was developed by Zong et al. (2005) to mark recombination events in both mitotic and postmitotic cells [24]. The MADM method enables in vivo analysis of gene function with simultaneous labeling and gene knockout at a single-cell level. Zong et al. used this method for conditional gene knockout and lineage analysis by showing that granule cell progenitors produce lineage-committed cells that project to specific areas of the cerebellar cortex. The MADM system is designed such that two reciprocally chimeric genes, green fluorescent protein (GFP) and red fluorescent protein (RFP) are knocked in on identical loci on homologous chromosomes. The N-terminus and C-terminus of the genes are separated by an intron containing a loxP site. Upon Cre activation, the Cre recombinase enzyme excises loxP sites, which allows for recombination between chromosomes after DNA replication. Upon G2-X segregation, two daughter cells are generated, with each cell expressing a single fluorescent protein (GFP or RFP). The interchromosomal recombination can result in indelible labeling of the daughter cells, and all of their progeny, with a single color, which facilitates investigating cell proliferation by examining the size of clones (Figure 1). Furthermore, quantification of GFP^+^ and RFP^+^ cells can reveal whether divisions are symmetric or asymmetric. In the case of symmetric divisions, GFP^+^ cells and RFP^+^ cells will be present in equal numbers, whereas in asymmetric divisions, one population may be present in greater numbers, pointing to the original cell as a potential progenitor [18]. In mice models in which the MADM transgenes are coupled to a cardiomyocyte-specific promoter, Cre recombination can unambiguously label cardiomyocytes that are proliferating.

### 2.2. Using MADMs to Study Cardiomyocyte Proliferation during Development

The use of MADMs in the heart was first described by Ali et al. [25]. In order to validate the model in the heart, they used an HprtCre^+/−^;MADM-11^GT/TG^ mouse, to investigate cardiomyocyte clonal expansion during embryonic development and after myocardial injury in adult hearts. They also knocked in the reconstituted GFP and RFP genes into the Hipp11 locus and found no single-labeled cells, suggesting that there is no silencing of the MADM transgene. 

**Figure 2:**
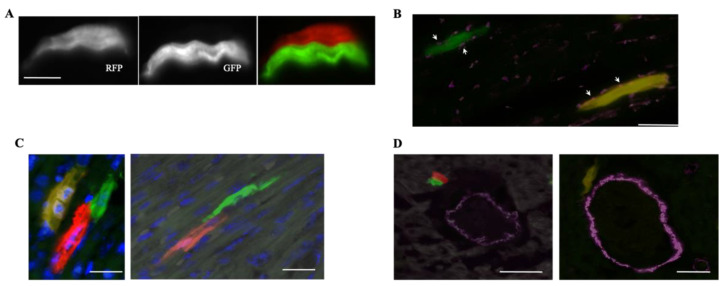
The utility of MADMs in studying cardiogenesis during development: (**A**) MADMs recombination in the Myh6^CreERT2^;MADM-11^GT/TG^ model generates two single-labeled RFP^+^ and GFP^+^ cardiomyocytes. (Scale bar, 15 μm) [25]; (**B**) staining for connexin43 (white arrows) reveals a GFP^+^ cell integrating into the myocardium (scale bar, 5 μm) [25]; (**C**) single-labeled RFP^+^ and GFP^+^ cardiomyocytes (generated by the β-actin^CreER^;MADM-11^GT/TG^ model) exhibiting intimate end-on contact (scale bars, 10 μm) [25]; (**D**) in the β-actin^CreER^;MADM-11^GT/TG^ model, cardiomyocytes and smooth muscle cells exhibit different MADM labeling (scale bars, 20 μm) [25].

In order to study cardiomyocyte regeneration, the MADM transgene was coupled with a cardiomyocyte-specific marker, alpha-myosin heavy chain (Myh6), to create the Myh6^CreERT2^;MADM-11^GT/TG^ mouse. Upon administration of exogenous tamoxifen (TM), MADM recombination could occur in cells that express Myh6. When a 2 mg TM dose was administered for one week to newborn pups, hearts analyzed at postnatal day 12 (P12) were found to have a labeling efficiency of 0.78%, with 11% of labeled cells being single-labeled (Figure 2A). There was a lack of large clones, with the occasional presence of two-cell clones, suggesting that cardiomyocytes undergo limited proliferation during postnatal development. Equivalent numbers of GFP^+^ and RFP^+^ cells were present, indicating the symmetric nature of cardiomyocyte division. MADM recombination was then induced at 1 month of age, and when hearts were analyzed, only 6% of labeled cells were single-labeled, demonstrating that the proliferative capacity of cardiomyocytes decreased with aging. Using sparse labeling of cells, they observed that cells were often noncontiguous, meaning that cardiomyocytes separate from each other after division. Connexin43 staining also demonstrated that single-labeled cells resulting from the division were able to integrate into the existing myocardial network (Figure 2B). When TM was administered to mice at embryonic day 13.5 (E13.5), large clones of labeled cells were identified, confirming that there is extensive cardiomyocyte proliferation during embryonic development. 

Ali et al. also recorded proliferation events in a β-actin^CreER^;MADM-11^GT/TG^ mouse model that allows for MADM recombination in any cell, including progenitors and mature cardiomyocytes. They found similar results to their Myh6^CreERT2^;MADM-11^GT/TG^ model and observed parity of GFP^+^ and RFP^+^ cells, further suggesting a low proliferative capacity of cardiomyocytes during development, with symmetric divisions (Figure 2C). Particularly, neither cardiomyocyte progenitor genes (Mesp1, Isl1, or Tbx5) nor clusters of endothelial cells, VSMCs, or cardiomyocytes were found, suggesting that adult cardiomyocytes are generated from pre-existing cardiomyocytes and not other cell types (Figure 2D). These data provide direct evidence to refute previous controversial studies that suggested the presence of cardiac stem/progenitor cells in the adult mammalian heart. 

Using an innovative approach, Nguyen et al. coupled MADMs to a conditional gene knockout, which allowed them to directly determine the contribution of specific genes to cardiogenesis in the postnatal heart [26]. They used a Myh6^mERcremER^ mouse model crossed with a MADM-11^GT/TG^ mouse and Hoxb13^fl/fl^ mouse to show that genetic deletion of Hoxb13 resulted in a significant increase in single-positive RFP and GFP cells. The MADM mouse model, crossed with a double-induced knockout of Meis1 and Hoxb13 also demonstrated a significant increase in single-positive RFP and GFP cells, compared with control.

### 2.3. Using MADMs to Study Clonal Regeneration during Disease

**Figure 3:**
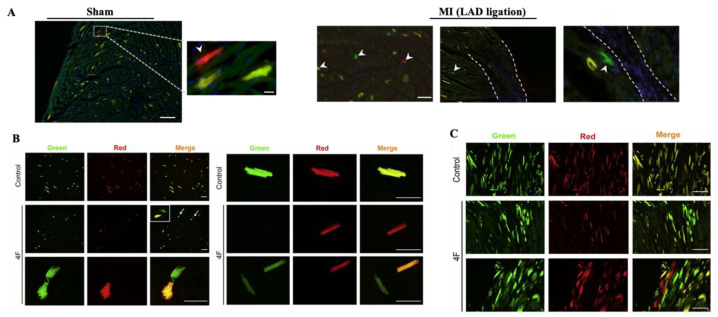
The utility of MADMs in studying cardiogenesis in response to acute ischemic injury: (**A**) white arrowheads point to proliferating RFP^+^ and GFP^+^ cardiomyocytes in Sham vs. MI, induced by LAD ligation, in the Myh6^CreERT2^;MADM-11^GT/TG^ model. There was no significant increase in proliferation from Sham to MI (scale bar, 100, 20, 100, 10 μm, from top panel to bottom panel) [25]; (**B**) immunofluorescence images showing single-positive RFP^+^ and GFP^+^ cells in the αMHC^mERcremER^;MADM-11^GT/TG^ model post-4F treatment [27]; (**C**) immunofluorescence imaging from the cTnT^Cre^;MADM-11^GT/TG^ model shows a significant increase in RFP^+^ and GFP^+^ cells post-4F treatment. The high number of single-positive cells can be attributed to the cTNT model labeling cells with a higher labeling efficiency and being active in the proliferative period before birth [27]; (**B**,**C**) adapted with permission from Ref. [27]. Copyright 2018, Copyright Elsevier.

Ali et al. used their β-actinCreER model to evaluate the potential contribution of progenitors in cardiac regeneration following MI [25]. TM was administered to 8-week-old mice, followed by ligation of the left anterior descending artery (LAD), to induce experimental MI. They found very low numbers of single-labeled cardiomyocytes in the infarct and peri-infarct regions, indicating that there is little cardiomyocyte division post-MI. Similarly, in the Myh6^CreERT2^;MADM-11^GT/TG^ model, there were low numbers of RFP^+^ and GFP^+^ single-labeled cells post-MI, compared with sham, indicating that pre-existing cardiomyocytes generate cardiomyocytes postinjury but with very little efficiency (Figure 3A). 

Several studies have suggested that enforced expression of certain cell cycle genes may induce cardiomyocytes to undergo proliferation. Mohamed et al. utilized an αMHC^mERcremER^;MADM-11^GT/TG^ mouse model to lineage trace cardiomyocyte proliferation post-MI in response to overexpression of the combination of cyclin-dependent kinase 1 (CDK1), cyclin-dependent kinase 4 (CDK4), cyclin B1, and cyclin D1 with adenovirus treatment (herein referred to as 4F) [27]. They found that the percentage of single-labeled cardiomyocytes increased significantly in response to 4F viral treatment after MI (Figure 3B). Cell cycle markers EdU and PH3 were present in single-labeled cardiomyocytes, and proliferating cardiomyocytes were present at the border and infarct zones. The number of single-positive cells in the control group was similar to the numbers reported by Ali et al. [25]. Interestingly, the percentage of yellow (double-labeled) cells remained similar between the control and 4F treatment. This suggests that the single-labeled cells increased in terms of both absolute number and proportion to all the cells in which MADM recombination occurred. This further attests to the finding that 4F treatment stimulates cardiomyocyte proliferation post-MI. A cTnT^Cre^;MADM-11^GT/TG^ model was also used to validate their findings in a model in which cardiomyocytes are labeled during early development. In these mice, the cardiac troponin-T (cTnT) gene is active during the proliferative period and has a higher labeling efficiency than that in the previous model; thus, in the control group, 10% of cells were single-labeled, while in the 4F group, 50% of cells were single-labeled (Figure 3C). 

Lastly, Magadum et al. studied the effect of introducing the Pkm2 gene into the heart to stimulate CM proliferation post-MI [28]. In contrast to previous MADM models, they used delivery of modified Cre RNA (modRNA) into a MADM-ML-11^GT/TG^ mouse, rather than using a Cre-expressing mouse, which is faster and more cost-effective. They performed a LAD ligation to simulate MI and administered an injection of cardiomyocyte-specific Pkm2 modRNA or Luciferase modRNA to label cells. Based on their results, 14 days post-MI, hearts treated with Pkm2 showed a higher percentage of single-labeled cardiomyocytes in the border and infarct zone than the luciferase-treated controls. Isolated CMs from hearts 14 days post-MI also showed more single-labeled cells. In addition, injection of Pkm2 modRNA into hearts 14 days post-MI revealed the presence of single-labeled cells, suggesting that Pkm2 can stimulate cardiogenesis post-MI.

### 2.4. Limitations

The main advantage of the MADM system is that it uses a single chromosomal event to couple recombination and labeling, which unambiguously labels two daughter cells with distinct colors. The single-color labeling allows for differentiation between karyokinesis and cytokinesis, as well as for the determination of whether clones are generated symmetrically or asymmetrically. The generation of single-colored clones occurs through G2-X segregation, which marks the birth date of individual cells and allows researchers to determine the parent cell that generated individual clones [24]. These features make MADMs particularly useful in stem cell and cancer studies, as tracking the size of clones can point to certain cell populations as progenitor or “origin” cells. As demonstrated above, MADMs can be used to reveal sources of cardiomyocyte proliferation both during development and in response to injury.

A large limitation of MADMs is that it relies on interchromosomal recombination rather than intrachromosomal recombination, which has a lower recombination efficiency [24]. However, when studying cardiac regeneration, this is not necessarily a limiting factor because sparse labeling of cells is advantageous in the context of lineage tracing and clonal analysis studies. In addition, MADM mouse models generally require extensive breeding schemes to generate models with three or more genes (gene of interest, Cre, MADM) [29]. In cardiomyocytes, different modes of mitosis may complicate recording clonal events through MADMs. MADM recombination generates single-labeled cells under only G2-X segregation, but under G2-Z segregation, recombination produces one double-labeled and one unlabeled cell, while under G0 recombination, a yellow cell is produced [24]. However, studies must only consider G2-X segregation events because they can be traced unambiguously, which could thereby lead to the underestimation of proliferating cells. Despite this limitation, reports using MADMs have corroborated previous studies that used other methods of detecting cell proliferation. In their original MADM study, Zong et al. reported that the distribution of granule cell clones was similar to those reported by an earlier clonal analysis study, using retroviral infection in chick embryos [24,30]. In addition, the results reported by Ali et al. supported a previous report from Senyo et al., which used mass spectrometry to show that cardiomyocytes renew cardiomyocytes after birth in mice [10,25]. Further research is required, however, to confirm how accurately the MADM system can estimate proliferation in various organs. Lastly, until recently, the MADM system could only be applied to chromosomes 6, 7, 11, and 12, which only allowed for analysis of less than 25% of genes [31]. Contreras et al. recently expanded the MADM cassettes to all 19 mouse autosomes, which allows future studies to utilize this system for other genes [31].

## 3. Rainbow Reporter System

### 3.1. Background and Methodology

The rainbow reporter system is a stochastic model that enables lineage tracing through the use of fluorophore-mediated cell labeling. When used in conjunction with the Cre/LoxP system, the rainbow reporter can label tissue-specific cells in order to trace their fate and proliferation over a given period of time. The progeny of the labeled parental cell continues to express the same fluorescent protein, making the rainbow model a useful tool for retrospective, in vivo lineage-tracing studies.

Rainbow is a lineage-tracing system that was developed based on Brainbow technology, which was first engineered by Livet et al. in 2007 to visualize synaptic circuits through neuron labeling [32]. The Brainbow system utilizes Cre-mediated recombination and consists of a cassette of three fluorescent proteins inserted into the Thy1 gene, which is known to be highly expressed in many neural cell types. The newest version of this system, Brainbow 3.0–3.2, uses Cre technology to excise DNA flanked by loxP sites in order to express one of three farnesylated fluorescent proteins in recombinant cells [33]. Brainbow was the first iteration of an in vivo retrospective lineage-tracing model and inspired the development of other labeling techniques for different cell types. The initial adaptation of this technology for application in cardiomyocytes was first used in zebrafish to assess their proliferative capacity and distinct contributions to chamber morphology. Gupta et al. employed the Brainbow system to investigate the role of clonal expansion in cardiac morphogenesis by characterizing the proliferative behaviors of cardiomyocytes. The Brainbow 1.0 L was crossed with a Cre line to generate a *cmlc2*:*CreER*; *priZm* transgenic zebrafish that strictly labels differentiated cardiomyocytes upon tamoxifen administration. This model allowed the investigators to conclude that ventricular myocardium in the adult zebrafish develops in order from primordial to trabecular and cortical muscle, with clonal populations of expanding cardiomyocytes at each stage [34].

Early models also include the *amhc*:*CreER*; *priZm* and *vmhc*:*CreER*; *priZm* zebrafish developed by Zhang et al. [35]. They developed inducible Cre transgenic lines driven by the atrial (amhc) or ventricular myosin heavy chain (vmhc) genes and crossed with a Brainbow reporter strain to specifically mark atrial or ventricular cardiomyocytes and their daughter cells. Foglia et al. used the *amhc*:*CreER*; *priZm* and *vmhc*:*CreER*; *priZm* zebrafish models to demonstrate that cardiomyocytes undergo clonal expansion to contribute to atrial and ventricular chamber growth [36]. Further studies have altered this existing technology and applied it to murine models using the rainbow reporter system.

**Figure 4:**
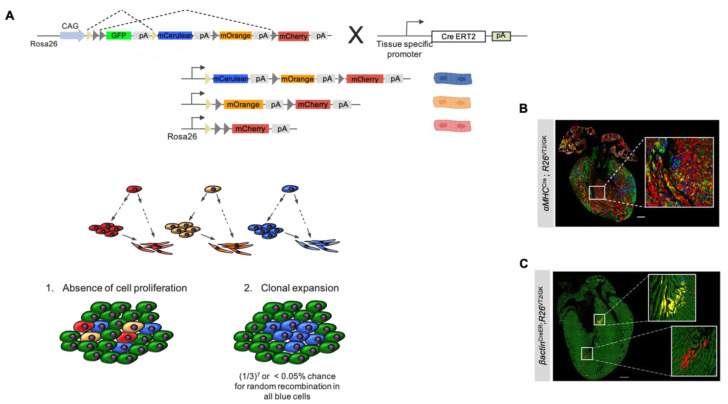
Clonal expansion of labeled cardiac cells using rainbow: (**A**) schematic representation of the rainbow reporter system in a Cre/LoxP transgenic mouse and diagram illustrating the difference between the absence and presence of clonal expansion in a population of rainbow-labeled cells; (**B**) Fluorescent light microscope image of an αMHC^Cre^;R26^VT2/GK^ longitudinal mouse heart section at P1. Labeled *αMHC*-expressing cardiomyocytes exhibit single-cell clones of various colors, without obvious clusters representative of clonal expansion (scale bar 50 μm) [38]; (**C**) fluorescent light microscope image of βactin^CreER^;R26^VT2/GK^ E10.5 heart. Clonal expansion was observed in βactin-expressing labeled cells, shown in outlined yellow and red clustered segments (scale bar 100 μm) [38].

The rainbow system depends on Cre-mediated recombination to mark target cells with one of three fluorescent labels. The following four genes are inserted in the Rosa26 (R26) locus of rainbow mice: GFP, mCerulean, mOrange, and mCherry (Figure 4A). The rainbow mouse (hereafter termed R26^VT2/GK^), when crossed with an inducible mouse (i.e., CreER line), allows for temporal control of recombination events, which results in the permanent expression of one of the fluorescent genes in the targeted cell type. Upon administration, tamoxifen binds to the estrogen receptor fused to Cre recombinase, which in turn activates the enzyme. The activated Cre recombinase excises a random pair of loxP sites flanking the region of DNA just preceding one of the fluorescent genes, causing irreversible expression of the fluorescent protein in the affected cell and its progeny [37]. In the absence of a recombination event, all cells and their progeny will express the default GFP. The rainbow system can successfully label a distinct cell type on the condition that CreER is expressed under the promoter sequence of a tissue-specific gene. For lineage tracing and clonal analysis of cardiomyocytes, the rainbow transgenic mouse can be crossed with a CM-specific promoter (i.e., αMHC or cTnT), such that tamoxifen administration can induce fluorescent labeling in only cardiomyocytes. While the amount of tamoxifen can control the intensity of cell labeling, the timing of administration has a temporal influence on the labeled cells. 

The arbitrary nature of the four-color rainbow reporter system minimizes the likelihood of neighboring cardiomyocytes expressing the same fluorescent marker. This allows for retrospective lineage tracing of clones to parent cells to be carried out with ease since clusters will likely be colored with different fluorescent labels. The utility of rainbow mouse models in cardiac tissue was first demonstrated by Sereti et al. in 2018 in an effort to characterize the proliferative capacity of cardiomyocytes during development, in adults, and after myocardial injury [38]. While this paper used the rainbow model as a method to investigate cardiomyocyte generation and regeneration, it also provided an overview of how this reporter system can provide a strong foundation for lineage tracing in the presence or absence of clonal expansion. Two neighboring, recombinant cells can express different colors; nevertheless, all progeny of each differentially labeled cell will fluoresce with the same marker; the daughter cells of a single progenitor are, therefore, easily identified, as they appear as a localized cluster of same-colored cells. Another unique aspect of the rainbow reporter system lies in its ability to quantify the proliferative capacity of cardiomyocytes in different regions of tissue. In the event of proliferation in the labeled cells, in vivo analysis will reveal clusters of multiple, same-colored cells (Figure 4A,C). If clonal expansion does not occur, the colored cells will not be found in localized groups and will instead be sparsely scattered throughout the cardiac tissue (Figure 4A,B). 

Previous studies mostly relied on two-dimensional quantification methods to estimate the extent of cell proliferation. However, this may introduce inaccuracies since the depth of cell clusters cannot be accounted for using cross-sections. Recent advances in tissue clearing allow for clear visualization of intrinsically complex organs, such as the heart, with unprecedented resolution. In order to accurately quantify the volume of each clone labeled by the rainbow system, the CLARITY technique can be used prior to light-sheet imaging [39]. CLARITY uses hydrogel embedding and membrane lipid removal to transform the intact organ into an optically translucent but structurally preserved tissue for light-sheet fluorescence or confocal microscopy [40]. The advantage of using CLARITY in rainbow hearts is its high-resolution imaging and ability to accurately quantify three-dimensional clone sizes with precise anatomical localization. Due to the robust fluorescent signals in the rainbow-labeled cells, fluorescence or confocal microscopy can be used to visualize labeled cells, and there is no need for immunostaining of the reporter proteins.

Rainbow technology has previously been used to quantify the proliferative capacities of cardiomyocytes, as well as various other cell types. Through rainbow-mediated cell labeling, researchers have been able to identify a dermal lineage with intrinsic fibrogenic potential, isolate mesothelial precursors, giving rise to smooth muscle cells and fibroblasts, investigate the cell dynamics of premalignant intestinal stem cells, disprove the presence of proliferating oocytes in postnatal mice, and characterize the changes in podocytes following kidney injury [41,42,43,44,45]. While prior studies have highlighted the diverse applicability of the rainbow reporter system, it has been used in the cardiovascular system to demonstrate a robust proliferation of cardiomyocytes during development, which significantly diminishes in adults.

### 3.2. Using Rainbow to Analyze Cardiomyocyte Proliferation during Development

**Figure 5:**
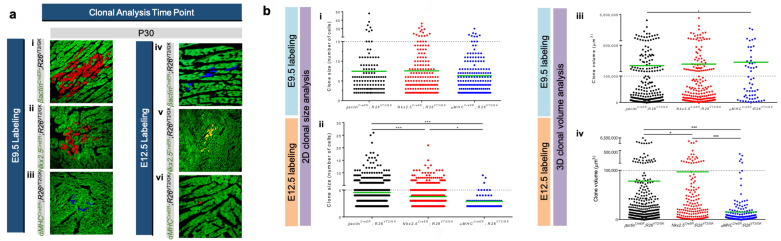
Clonal Expansion in the in vivo developing heart: (**a**) confocal microscope images of labeled clones in (i,iv) βactin^CreER^;R26^VT2/GK^, (ii,v) Nkx2.5^CreER^;R26^VT2/GK^ and (iii,vi) αMHC^CreER^;R26^VT2/GK^ mouse hearts at P30. In this study, 4-OHT administration and subsequent cell-labeling were completed at (i–iii) E9.5 or (iv–vi) E12.5. Clonal clusters were more evident in (i,ii,iv,v) cardiac progenitor cells compared with (iii,vi) cardiomyocytes [38]; (**b**) analysis of (i,ii) labeled clone sizes and (iii,iv) labeled clone volumes at E9.5 and E12.5 in βactin^CreER^;R26^VT2/GK^, Nkx2.5^CreER^;R26^VT2/GK^, and αMHC^CreER^;R26^VT2/GK^ mouse hearts, respectively. (i,iii) similar clone sizes and volumes were observed in cardiac progenitors and cardiomyocytes at E9.5. (ii,iv) Decreased clone volume in cardiomyocytes compared to cardiac progenitor cells at E12.5, with (iv) αMHC-expressing cardiomyocyte clone volume showing the most drastic decrease [38].

Sereti et al. used the rainbow reporter system to characterize cardiomyocyte proliferation at different stages of cardiovascular development [38]. This study used αMHC^CreER^;R26^VT2/GK^ mice to label cardiomyocytes in embryonic mice and analyzed the postnatal hearts after tamoxifen injections given on embryonic day 9.5 (E9.5) and E12.5. At E9.5, labeled αMHC-expressing cardiomyocytes exhibited similar clone size and volume compared to Nkx2.5^CreER^;R26^VT2/GK^ and βactin^CreER^;R26^VT2/GK^ mice, which fluorescently mark cardiac progenitor cells and all cell types, respectively. At E12.5, however, a dramatic decrease in clonal volume was observed in αMHC^CreER^;R26^VT2/GK^ mice when compared with E9.5 αMHC-labeled cells (Figure 5b). Furthermore, at E12.5, βactin-expressing cells and Nkx2.5-expressing cardiac progenitors exhibited a minor decrease in proliferation, whereas αMHC cells showed a striking decrease, suggesting that as cardiomyocytes mature, their proliferative capacity decreases in comparison to cardiac progenitors at the same developmental stage (Figure 5a). The findings of this study suggest that even during in utero development, maturing cardiomyocytes exhibit a lower proliferative capacity, compared with cardiac progenitors, and this capacity continues to diminish postnatally.

The rainbow reporter system has proven to be a useful technique for retrospective lineage tracing in vivo, but also as a functional tool to track the differentiation of cardiomyocytes in vitro. This was demonstrated by El-Nachef et al. in 2020, in a study in which the authors generated a rainbow human-induced pluripotent stem cell (hiPSC) line and tracked the cells’ differentiation into cell types from all germ layers, including cardiomyocytes [46]. Rainbow hiPSCs were created by inserting a cassette of three fluorescent genes flanked with lox sequences, all downstream from a CAG promoter in the AAVS1 gene locus. The hiPSCs were engineered such that upon Cre treatment, irreversible recombination takes place and the cell membranes of the labeled cell, and its progeny express one of three fluorescent proteins. The investigators used the rainbow hiPSCs to longitudinally monitor their differentiation into various cell types while at the same time interrogating their precursor–progeny relationship. Individually labeled cardiac progenitors were tracked through their differentiation into mature cardiomyocytes (marked by the presence of hypertrophy and expression of cTnT). In a later study using rainbow technology, it was proven that transplanted human pluripotent stem cell-derived cardiomyocytes (hPSC-CM) exhibit clonal expansion both in vivo and in vitro. Rainbow hPSCs were generated, then transduced with cTnT-driven Cre, and immunostained for confirmation of differentiation into cardiomyocytes [47]. hPSC-CMs demonstrated clonal expansion by day 14 in vitro. Then, 14-day hPSC-CMs were injected into rat hearts and showed continuous clonal expansion up to 6 weeks after engraftment. The results confirm that in the context of cardiomyocytes, the rainbow reporter system can stably label and track cells from their early stages as hiPSCs through differentiation into their mature form, as well as their clonal expansion.

### 3.3. Using Rainbow to Analyze Cardiomyocyte Proliferation during Disease

**Figure 6:**
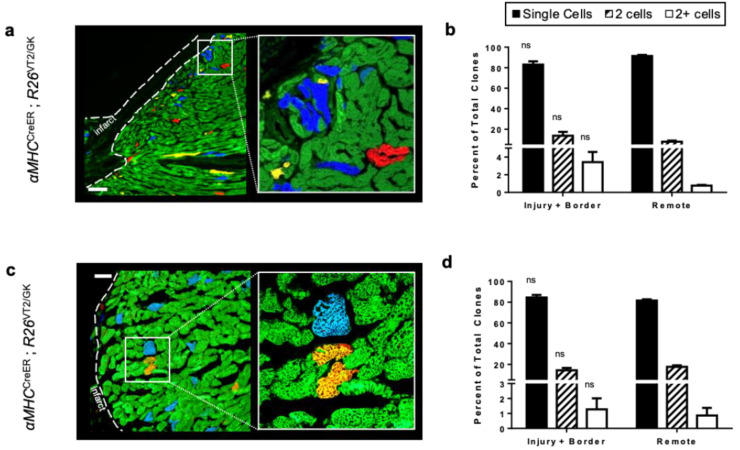
Clonal Expansion in the in vivo diseased heart. Confocal microscope images depicting rainbow-labeled cardiomyocytes in neonatal mouse hearts in response to LAD injury at (**a**) P1 and at (**c**) 8 weeks of age. Analysis was conducted 21 days postinjury [38]; (**b**,**d**) quantification of cardiac clones following LAD injury. No significant (ns) differences were observed, suggesting that cardiomyocyte generation is due to cardiomyocyte division rather than clonal expansion after injury [38].

The rainbow system has also proven to be a useful lineage-tracing tool in studying cardiomyocyte proliferation in cardiovascular disease. Sereti et al. studied the effect of MI on cardiomyocyte proliferation in adult and neonatal mice using the rainbow reporter system [38]. Newborn αMHC^CreER^;R26^VT2/GK^ mice were injected with tamoxifen and then underwent LAD ligation 1 day later. Hearts were analyzed at 21 days postinjury, and it was observed that most of the cardiac tissue had regenerated with only a small amount of scar tissue remaining. Through confocal microscopic analysis of αMHC-labeled hearts, frequent cardiomyocyte clones were found in the infarct and border zones (Figure 6a,b). These findings confirmed that newly generated cardiomyocytes are derived from pre-existing cardiomyocytes. A similar experimental approach was performed in adult αMHC^CreER^;R26^VT2/GK^ mice: after injury, a greater area of scar formation and lack of large labeled cardiomyocyte clones were observed (Figure 6c,d). The clone quantification data demonstrated that neonatal hearts have a higher proliferative capacity in response to myocardial injury compared with adult hearts, suggesting that the ability for cardiomyocytes to proliferate significantly diminishes with aging on a continuous timescale.

The rainbow reporter system has many potential applications for future studies in cardiovascular research. One major area of interest is using stem/progenitor cell therapies to regenerate damaged myocardium after an ischemic cardiac injury. Due to the low regenerative capacity of adult cardiac tissue, the live cardiomyocytes remaining after a myocardial infarction do not proliferate to compensate for lost tissue. Instead, fibrosis replaces the necrotic tissue, which eventually leads to decreased cardiac function and heart failure [48]. Current research is investigating the ability to deliver exogenous cardiomyocytes or activate the proliferation of existing ones, to generate new functioning myocardial tissue and improve long-term heart function postmyocardial infarction [49]. The rainbow reporter system can be used to track transplanted cells postinfarct and follow their fate and proliferation at different time points during the wound healing process. Due to its ability to permanently label cardiomyocytes and their progeny, the rainbow system is a promising tool to characterize the efficacy of cell delivery by quantifying their proliferative capacities in injured adult hearts.

### 3.4. Rainbow vs. Other Multicolor Lineage-Tracing Models

Other genetic mouse models with similar technologies have been previously used for in vivo lineage tracing. As mentioned in Section 3.1, the Brainbow system pioneered multicolor lineage-tracing models used for in vivo studies. Although engineered for neural tissue, this model has been widely used in various organ systems including the heart. One limitation of Brainbow is that due to the stochastic nature of the system, it is unable to differentially label every cell in a desired neural circuit, which could lead to difficulties with identifying specific neurons. While precise circuit labeling is crucial for neural network research, it is not as big of a concern for research regarding cardiomyocyte proliferation.

Another commonly used lineage-tracing model is the Confetti mouse system. This reporter model involves the insertion of the Brainbow 2.1 cassette, a neomycin roadblock (Neo^R^) cassette, and a CAGG promoter into the Rosa26 gene locus [50]. Following Cre-mediated activation, the Neo^R^ cassette is removed, resulting in the recombined cell fluorescing in one of three possible colors. Snippert et al. first used Confetti as a multicolor lineage-tracing model to characterize homeostatic self-renewal control in Lgr5-expressing intestinal stem cells. Similar to rainbow, Confetti was engineered as a means to study a variety of tissues, including non-neural cell types while still using Brainbow technology. A limitation of this model is that the expression of a fluorophore is acquired through Cre-mediated inversion instead of deletion. This diminishes the integrity of the reporter system, as the cell cannot be permanently labeled and is subject to change colors over time. While the Brainbow and Confetti systems both use transgenes, the rainbow system was generated by knocking in the fluorescent reporter genes into a constitutively active site, which results in more effective labeling. It is worth noting that the efficiency of all three systems largely depends on the tissue-specific Cre mouse model. It has been demonstrated that the rainbow system offers a more equal distribution of the three fluorescent proteins, hence making cell tracking more reliable.

### 3.5. Limitations

The rainbow reporter system has proven to be a useful technique for cardiovascular research, but it is not without limitations. While the random labeling nature of the rainbow model provides for multiple colors to be expressed within the same tissue, allowing for tracking of various groups of cell proliferation, this is also a limitation of the model since one cannot control the color assignments for each labeled cell. Hence, there is always a small chance that adjacent cardiomyocytes may be labeled identically, creating difficulty in making distinctions between the proliferation of the two cells and their same-color progeny. To address this, a future direction for the rainbow system is manipulating the genetic model to include more fluorescent genes rather than only the current three. This would minimize the chance of two adjacent cardiomyocytes being labeled with the same color and would result in increased confidence in data interpretation. One approach could be incorporating multiaddressable genome-integrative color (MAGIC) markers technology with the rainbow reporter system. The MAGIC markers toolkit uses a variety of fluorescent proteins to label cells and also assigns these fluorophores to one of the various possible subcellular locations [51]. Loulier et al designed multiple MAGIC marker cassettes that achieve cytosolic, nuclear, membranous, or mitochondrial fluorescent labeling of designated cells, which was successfully demonstrated in the neural tissue of a mouse model. The ability to mark cells using two different labeling elements further minimizes the possibility of labeling adjacent cardiomyocytes in the same way. Additionally, in order to increase confidence in distinct cell-labeling through the rainbow model, one approach is to incorporate multiple copies of the rainbow cassette in the transgenic mouse model, as demonstrated in the original Brainbow model by Livet et al. [32].

Another important aspect to account for when utilizing the rainbow reporter system in vivo is that the Cre/LoxP mouse line must be tightly regulated with high tissue specificity for the desired cell type to be faithfully labeled. The appropriate tamoxifen administration must also be considered, as its dosage and duration vary depending on the mouse line [52]. Both these factors must be optimized to promote the most efficient and apparent labeling of the designated cell types using the rainbow reporter system.

## Data Availability

Not applicable.
